# Time course of pulmonary burden in mice exposed to residual oil fly ash

**DOI:** 10.3389/fphys.2014.00366

**Published:** 2014-09-25

**Authors:** Giovanna Marcella Cavalcante Carvalho, Lilian Katiê da Silva Nagato, Sheila da Silva Fagundes, Flávia Brandão dos Santos, Andrea Surrage Calheiros, Olaf Malm, Patricia Torres Bozza, Paulo Hilário N. Saldiva, Débora Souza Faffe, Patricia Rieken Macedo Rocco, Walter Araujo Zin

**Affiliations:** ^1^Laboratory of Respiration Physiology, Carlos Chagas Filho Institute of Biophysics, Universidade Federal do Rio de JaneiroRio de Janeiro, Brazil; ^2^Laboratory of Immunopharmacology, Department of Physiology and Pharmacodynamics, Oswaldo Cruz Institute, Fundação Oswaldo CruzRio de Janeiro, Brazil; ^3^Laboratory of Radioisotopes, Carlos Chagas Filho Institute of Biophysics, Universidade Federal do Rio de JaneiroRio de Janeiro, Brazil; ^4^Laboratory of Experimental Air Pollution, Department of Pathology, School of Medicine, Universidade de São PauloSão Paulo, Brazil; ^5^Laboratory of Macromolecular Metabolism Firmino Torres de Castro, Carlos Chagas Filho Institute of Biophysics, Universidade Federal do Rio de JaneiroRio de Janeiro, Brazil; ^6^Laboratory of Pulmonary Investigation, Carlos Chagas Filho Institute of Biophysics, Universidade Federal do Rio de JaneiroRio de Janeiro, Brazil

**Keywords:** air pollution, residual oil fly ash (ROFA), lung mechanics, pulmonary histology, lung injury, ROFA composition

## Abstract

Residual oil fly ash (ROFA) is a common pollutant in areas where oil is burned. This particulate matter (PM) with a broad distribution of particle diameters can be inhaled by human beings and putatively damage their respiratory system. Although some studies deal with cultured cells, animals, and even epidemiological issues, so far a comprehensive analysis of respiratory outcomes as a function of the time elapsed after exposure to a low dose of ROFA is wanted. Thus, we aimed to investigate the time course of mechanical, histological, and inflammatory lung changes, as well as neutrophils in the blood, in mice exposed to ROFA until 5 days after exposure. BALB/c mice (25 ± 5 g) were randomly divided into 7 groups and intranasally instilled with either 10 μL of sterile saline solution (0.9% NaCl, CTRL) or ROFA (0.2 μg in 10 μL of saline solution). Pulmonary mechanics, histology (normal and collapsed alveoli, mononuclear and polymorphonuclear cells, and ultrastructure), neutrophils (in blood and bronchoalveolar lavage fluid) were determined at 6 h in CTRL and at 6, 24, 48, 72, 96, and 120 h after ROFA exposure. ROFA contained metal elements, especially iron, polycyclic aromatic hydrocarbons (PAHs), and organochlorines. Lung resistive pressure augmented early (6 h) in the course of lung injury and other mechanical, histological and inflammatory parameters increased at 24 h, returning to control values at 120 h. Blood neutrophilia was present only at 24 and 48 h after exposure. Swelling of endothelial cells with adherent neutrophils was detected after ROFA instillation. No neutrophils were present in the lavage fluid. In conclusion, the exposure to ROFA, even in low doses, induced early changes in pulmonary mechanics, lung histology and accumulation of neutrophils in blood of mice that lasted for 4 days and disappeared spontaneously.

## Introduction

Many studies associate events of urban air pollution with significant health effects on the exposed population, including morbidity and mortality due to cardiopulmonary diseases or lung cancer (Dominici et al., 2006; Fajersztajn et al., 2013). These outcomes have been observed even at pollution levels below current national and international ambient air quality health standards (Lin et al., 1999).

Elevated levels of air pollution in São Paulo (Brazil) have been associated with increased respiratory emergency visits, hospital admissions and even death among children and elderly people (Saldiva et al., 1994, 1995; Lin et al., 1999, 2004; Farhat et al., 2005; Atkinson et al., 2014). These results are in accordance with experimental data from air pollution studies in São Paulo. Acute exposure to diesel and traffic-derived particles impairs lung impedance, pulmonary inflammation and histology in mice (Pereira et al., 1995, 2011; Laks et al., 2008; Mazzoli-Rocha et al., 2008; Zanchi et al., 2010; Riva et al., 2011; Zin et al., 2011). In addition, long-term mice exposure to traffic-derived particulate matter (PM) yielded worse pulmonary function, bronchial/alveolar lesion, lung macrophage influx, and oxidative stress (Mazzoli-Rocha et al., 2014), secretory cell hyperplasia and ultrastructural ciliary alterations of the airway epithelium (Saldiva et al., 1992a), compromised respiratory defenses (Lemos et al., 1994), as well as cardiopulmonary oxidative damage (Damiani et al., 2012).

Residual oil fly ash (ROFA) consists of PM produced by oil-burning systems and is rich in transition metals. It has been used in murine models as a surrogate material to investigate the responses to PM inhalation (Dreher et al., 1997; Kodavanti et al., 1998). ROFA administration via intratracheal/intranasal instillation and aerosol inhalation disclosed functional and structural alterations such as acute lung injury, alveolar septal thickening, increased cellularity and lung inflammation (Dreher et al., 1997; Gavett et al., 1997, 1999; Ghio et al., 2002; Hamada et al., 2002; Kodavanti et al., 2002). Additionally, ROFA exposure has been studied in association with chronic allergic pulmonary inflammation, cigarette smoke, and lung infection (Gavett et al., 1999; Antonini et al., 2002; Arantes-Costa et al., 2008; Biselli et al., 2011). We previously reported that chronic allergic mice exposed to ROFA show even higher hyperresponsiveness, bronchoconstriction and mast cell infiltration after methacholine challenge than those not exposed (Avila et al., 2011). However, so far the timeline of the lung alterations following a single exposure to ROFA particles has not been reported.

Thus, we aimed to investigate the time-dependency of lung impairment in animals that underwent a single exposure to ROFA, simulating the situation of someone visiting a polluted place for a day. For such purpose, we analyzed ROFA composition, lung mechanics, alveolar collapse, inflammatory cells recruitment, and pulmonary ultrastructure in different time points after exposure.

## Materials and methods

### Animals

All animals received humane care in compliance with the “Principles of Laboratory Animal Care” formulated by the National Society for Medical Research and the “Guide for the Care and Use of Laboratory Animals” prepared by the National Academy of Sciences, USA. The experiments were approved by the Ethics Committee on the Use of Animal, Health Sciences Center, Federal University of Rio de Janeiro (Protocol IBCCF 046).

Eighty-four BALB/c mice (6–7 weeks of age) obtained from the animal facilities of the Federal University of Rio de Janeiro, Brazil, were housed in plastic cages with absorbent bedding material and maintained on a 12-h daylight cycle. Food and water were provided *ad libitum*.

### Preparation

Forty-two mice (25–30 g) were randomly divided into 7 groups intranasally instilled with: sterile saline solution (0.9% NaCl, CTRL, *n* = 6) or ROFA (0.2 μg of ROFA in 10 μL of saline solution, *n* = 36). In CTRL group the experiments were done at 6 h after instillation, whereas in ROFA groups the mice were studied at 6, 24, 48, 72, 96, and 120 h after exposure (*n* = 6/group). Right before the instillation, the mice were anesthetized with sevoflurane and either saline or ROFA were gently instilled into their snouts with the aid of a precision pipette. They rapidly recovered after instillation. These animals were used for the measurement of pulmonary mechanics and histology. In another group of 42 mice submitted to the same protocol, inflammatory cells were counted in the blood and in the broncho-alveolar lavage fluid (BALF).

### ROFA composition

The ROFA was obtained from an incinerator located at the University Hospital, University of São Paulo, Brazil. The particles were digested in an HNO_3_–HClO_4_ mixture and then analyzed by flame atomic absorption spectroscopy (VARIAN AA1475, Varian, Inc., Palo Alto, CA, USA) to determine their elemental composition. ROFA was also analyzed by gas chromatography (GC-14B with automatic injector AOC-1400, Shimadzu Corp, Kyoto, Japan) and high performance liquid chromatography (RF-10 with fluorescence detectors, Shimadzu Corp, Kyoto, Japan) for organochlorine and polycyclic aromatic hydrocarbon (PAH) quantification, respectively. All analytical procedures above were determined as formerly described (Mazzoli-Rocha et al., 2008; Riva et al., 2011). The distribution of particle sizes was previously reported (Avila et al., 2011), and the average particle diameter amounted to 66.5 μm. It should be stressed that around 7.6% of ROFA particles presented an average diameter smaller than 10 μm, and about 2.1% were smaller than 2.5 μm (Avila et al., 2011).

### Pulmonary mechanics

At the aforementioned experimental times after instillation the animals were sedated (diazepam, 1 mg i.p.), anesthetized (pentobarbital sodium, 20 mg/kg i.p.), tracheotomized, and a snugly fitting cannula (0.8 mm i.d.) was introduced into the trachea. Then, the animals were paralyzed with pancuronium bromide (0.1 mg/kg i.v.), and ventilated (frequency of 100 breaths/min, tidal volume of 0.2 ml, and flow of 1 ml/s) with a constant-flow ventilator (Samay VR15, Universidad de la Republica, Montevideo, Uruguay). A positive end-expiratory pressure amounting to 2 cmH_2_O (Saldiva et al., 1992b) was applied to the expiratory line of the ventilator and the anterior chest wall was surgically removed. For the determination of pulmonary mechanics a 5-s end-inspiratory pause could be generated by the ventilator when needed.

A pneumotachograph (1.5 mm ID, length = 4.2 cm, distance between side ports = 2.1 cm) was connected to the tracheal cannula for the measurements of airflow (V′). Lung volume (V_T_) was determined by V′ signal integration. The equipment resistance (Req) including the tracheal cannula was calculated (Req = 0.12 cmH_2_O/mL/s) and found constant up to flow rates of 26 mL/s. The equipment resistive pressure (Pres,eq = Req·V′) was subtracted from pulmonary resistive pressure so that the present results represent intrinsic values. Transpulmonary pressure (PL) was measured proximally to the tracheal tube by a Validyne MP45-2 differential pressure transducer (Engineering Corp., Northridge, CA, USA). All signals were conditioned and amplified in a Beckman type R Dynograph (Schiller Park, IL, USA). Flow and pressure signals were also passed through low-pass 8-pole Bessel filters (902LPF, Frequency Devices, Haverhill, MA, USA) with the corner frequency set at 100 Hz, sampled at 200 Hz with a 12-bit analog-to-digital converter (DT2801A, Data Translation, Marlboro, MA, USA), and stored on a microcomputer. All data were collected using LABDAT software (RHT-InfoData Inc., Montreal, QC, Canada).

Lung resistive (ΔP1) and viscoelastic/inhomogeneous (ΔP2) pressures, total pressure drop (ΔPtot = ΔP1 + ΔP2), static elastance (Est), and elastic component of viscoelasticity (ΔE) were computed by the end-inflation occlusion method (Bates et al., 1985, 1988). Briefly, after end-inspiratory occlusion, there is an initial fast drop in PL (ΔP1) from the pre-occlusion value down to an inflection point (Pi) followed by a slow pressure decay (ΔP2), until an apparent plateau is reached. This plateau corresponds to the elastic recoil pressure of the lung (Pel). ΔP1 selectively reflects airway resistance in normal animals and humans and ΔP2 reflects stress relaxation or viscoelastic properties of the lung, together with a small contribution of time constant inequalities (Bates et al., 1988; Saldiva et al., 1992b). Lung static (Est) and dynamic elastances (Edyn) were calculated by dividing Pel and Pi by V_T_, respectively. ΔE was calculated as Est—Edyn (Bates et al., 1985, 1988). Pulmonary mechanics was measured 10–15 times in each animal. All data were analyzed using ANADAT data analysis software (RHT-InfoData Inc., Montreal, QC, Canada). The duration of the experiments approximated 30 min.

### Histological study

#### Light microscopy

A lower longitudinal laparotomy was done immediately after the determination of pulmonary mechanics, and heparin (1000 IU) was injected into the abdominal vena cava. Three minutes later the abdominal aorta and vena cava were sectioned, yielding a massive hemorrhage that quickly euthanized the animal. The trachea was clamped at end-expiration and the lungs were removed *en bloc*.

The left lung was quick-frozen by immersion in liquid nitrogen, fixed with Carnoy's solution (Nagase et al., 1992), and embedded in paraffin. Four-μm-thick slices were obtained by means of a microtome and stained with hematoxylin and eosin. Morphometry and cellularity index were evaluated with an integrating eyepiece with a coherent system with 100 points and 50 lines coupled to a conventional light microscope (Axioplan, Zeiss, Oberkochen, Germany). The point-counting technique was used across 10 random non-coincident microscopic fields to evaluate the fraction area of normal and collapsed airspaces and the amount of mononuclear (MN) and polymorphonuclear cells (PMN). Points falling on normal alveoli and collapsed airspaces were counted and divided by the total number of points in each microscopic field (200×). Points falling on MN and PMN cells were counted and divided by the total number of points falling on tissue area in each microscopic field (1000×) (Weibel et al., 1966). Two investigators, who were unaware of the origin of the coded material, examined the samples microscopically.

#### Transmission electron microscopy

To obtain a stratified random sample, three slices of 2 × 2 mm were cut from three different segments of the right lung (cranial, middle, and caudal lobes) and then fixed in 2.5% glutaraldehyde and 0.1 M phosphate buffer (pH = 7.4) for 60 min at −4°C. The slices were then rinsed in phosphate buffer, postfixed in 1% osmic tetroxide in phosphate buffer for 30 min, and rewashed three times in phosphate buffer. Finally, the slices were dehydrated in an acetone series and then placed in a mixture of 1:1 acetone:Epon overnight before embedding in Epon for 6 h. After fixation, the material was kept for 48 h at 60°C before undergoing ultramicrotomy for transmission electron microscopy (JEOL 1010, Tokyo, Japan).

### Evaluation of neutrophils in the blood and in the bronchoalveolar lavage fluid

At each experimental time, the animals were anesthetized with isoflurane and the tip of their tails were cut off to produce a blood smear. Neutrophil counts were determined in a Neubauer chamber by means of an optical microscope after dilution of blood samples in 2% acetic acid solution. The number of circulating neutrophils (100 cells counted/slide, 1000× magnification) was determined after differential cell counts on May-Grunwald-Giemsa stained blood smears. After blood sampling the mice were euthanized in a CO_2_ chamber and the alveolar lavage done. For such purpose, the trachea was cannulated and the lungs gently washed twice with 1 ml of phosphate buffered saline (pH = 7.4). Neutrophils were counted after cytocentrifugation (Shandon, East Grinstead, UK) and staining with Diff-Quick (Baxter Dade AG, Dunding, Germany). At least 100 cells were counted and the results expressed as number of cells/mL.

### Statistical analysis

SigmaStat 11.0 statistical software (SYSTAT, Chicago, IL, USA) was used. When percentage values were to be tested, they firstly underwent arcsine transformation. The normality of the data (Kolmogorov–Smirnov test with Lilliefors' correction) and the homogeneity of variances (Levene median test) were tested. Since in all instances both conditions were satisfied, One-Way ANOVA followed by Bonferroni *post hoc* test was used (when required) to assess differences between ROFA groups and CTRL mice. The significance level was set at 5% (*p* < 0.05).

## Results

ROFA analysis showed the presence of metal elements, such as copper, cadmium, chromium, nickel, manganese, lead, zinc and mainly iron (Table 1), and PAHs, such as naphthalene, acenaphthylene, fluorene, acenaphthene, antracene, flouranthene, phyrene, benzo[k]fluorantene, benzo[ghi]peryle (some with carcinogenic potencial: benzo[a]antracene, benzo[a]pyrene, Dbenzo[ah]antracene and ind[123cd]pyrene) (Table 2). Organochloride elements as g-hexachlorocyclohexane (g-HCH), endosulfan, dieldrin, op'-DDE (dichlorodiphenyl dichloroethylene), op'-DDT (dichlorodiphenyltrichloroethane), pp'-DDT were also present (Table 3).

**Table 1:** **Concentrations of metal elements in ROFA**.

**Metal**	**ppm (mean ± *SD*)**
Copper	5.64 ± 1.09
Cadmium	0.01 ± 0.00
Chromium	4.20 ± 0.71
Nickel	467.19 ± 9.75
Manganese	32.42 ± 4.60
Iron	12265.77 ± 2697.33
Lead	0.58 ± 0.18
Zinc	21.12 ± 1.34

*ROFA, residual oil fly ash; ppm, parts per million. Values are mean ± SD of three determinations*.

**Table 2:** **Polycyclic aromatic hydrocarbons in ROFA**.

**PAH**	**Concentration (mg/kg)**
Naphthalene	95.2
Acenaphthylene	155.6
Fluorene	2.6
Acenaphthene	67.8
Phenanthrene	ND
Anthracene	1.7
Fluoranthene	5.9
Phyrene	13.9
Chrysene	ND
Benzo[a]anthracene^*^	3.5
Benzo[b]fluorantene^*^	ND
Benzo[k]fluorantene	7.1
Benzo[a]pyrene^*^	2.8
DBenzo[ah]anthracene^*^	13.0
Benzo[ghi]peryle	1.5
Ind[123cd]pyrene^*^	1.7

Total PAH, polycyclic aromatic hydrocarbons in a 0.23 g sample; ROFA, residual oil fly ash; ND, not detectable;

* *polycyclic aromatic hydrocarbons with carcinogenic potential in mammals, as considered by the International Agency for Research on Cancer, USA*.

**Table 3:** **Organochloride in ROFA**.

**Orgnochloride**	**Concentration (ng/g)**	**Orgnochloride**	**Concentration (ng/g)**
G-HCH	121.7	PCB-118	ND
HCB	ND	PCB-138	ND
Heptachlor	ND	PCB-153	ND
Endosulfan	57.4	PCB-180	ND
Aldrin	ND	PCB-209	ND
Dieldrin	40.9	op'-DDE	391.3
Endrin	ND	pp'-DDE	ND
Hepta-epox	ND	pp'-DDD	ND
PCB-25	ND	op'-DDT	78.3
PCB-52	ND	pp'-DDT	139.1

*ROFA, residual oil fly ash; ND, not detectable; G-HCH, gamma-hexachlorocyclohexane; HCB, hexachlorobenzene; PCB, polychlorinated biphenyl; op', ortho position; pp', para position; DDE, dichlorodiphenyldichloroethylene; DDD, dichlorodiphenyldichloroethane; DDT, dichlorodiphenyltrichloroethane*.

**Figure 1:**
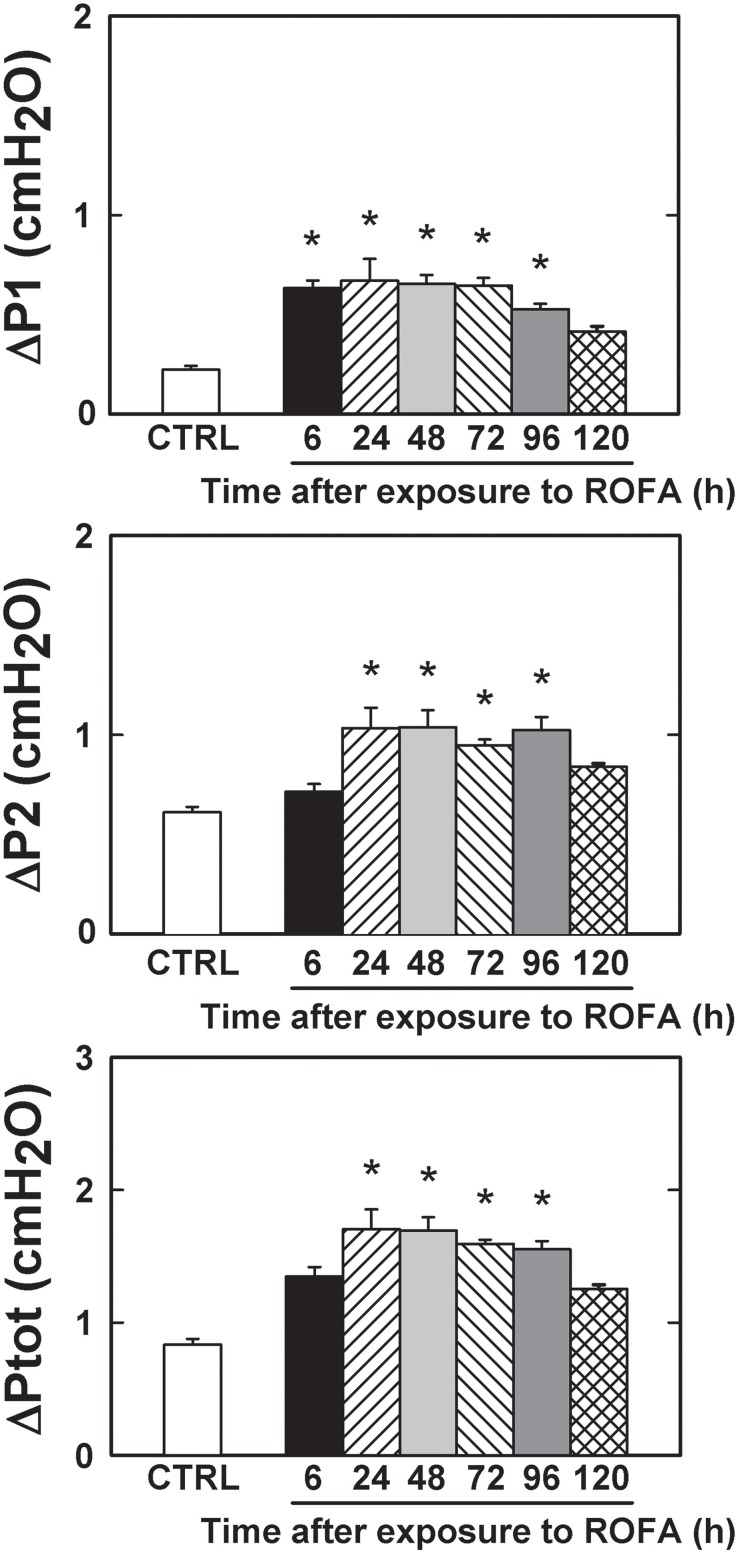
**Pressures used to overcome resistances in mice lung**. ΔP1, resistive pressure, ΔP2, pressure dissipated to overcome viscoelastic/inhomogeneous mechanical components and ΔPtot, total pressure variation. CTRL, mice instilled with saline solution (0.9% NaCl, measurements were done 6 h after exposure), and ROFA, animals that received residual oil fly ash (0.2 μg in 10 μL of saline solution). Measurements were done 6, 24, 48, 72, 96, and 120 h after exposure. Columns represent the average of 6 mice in each group, 10–15 determinations per animal. Bars represent SEM. ^*^Significantly different from CTRL (*p* < 0.05).

**Figure 2:**
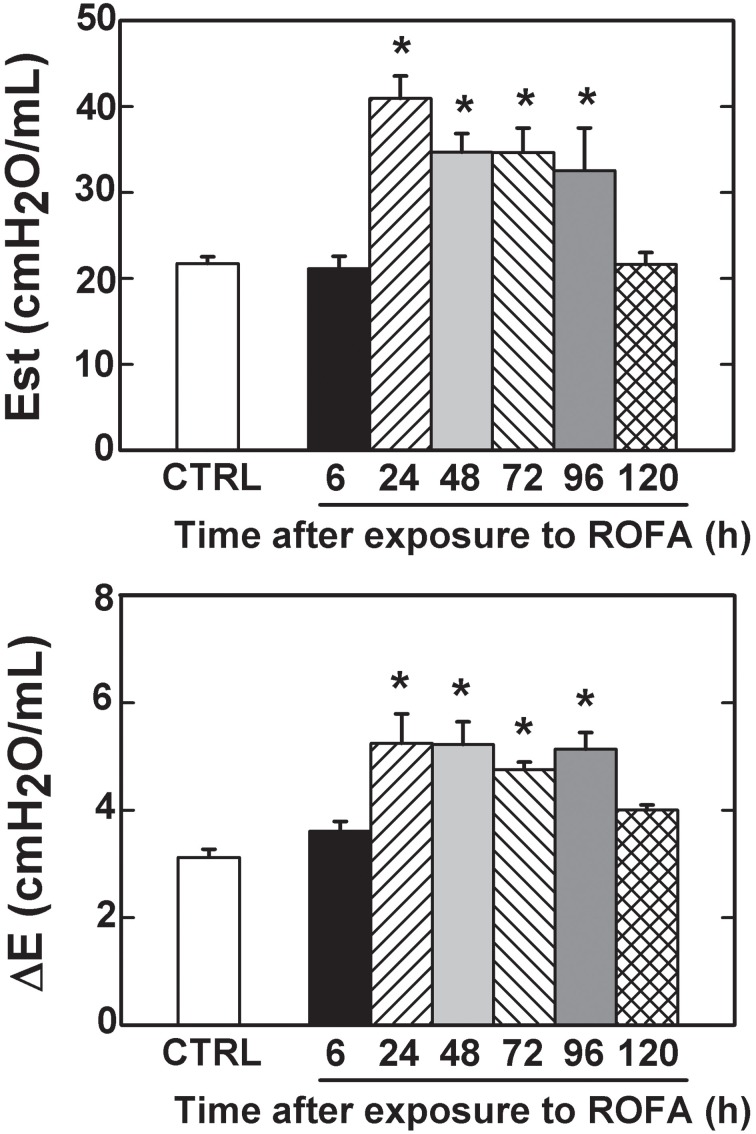
**Elastic components of lung mechanics in mice**. Est, static elastance and ΔE, elastic component of viscoelasticity. CTRL, mice instilled with saline solution (0.9% NaCl, measurements were done 6 h after exposure), and ROFA, animals that received residual oil fly ash (0.2 μg in 10 μL of saline solution). Measurements were done 6, 24, 48, 72, 96, and 120 h after exposure. Columns represent the average of 6 mice in each group, 10–15 determinations per animal. Bars represent SEM. ^*^Significantly different from CTRL (*p* < 0.05).

Flows and inspired volumes did not differ among groups. Figure 1 shows ΔP's, obtained in CTRL, ROFA6, ROFA24, ROFA48, ROFA72, ROFA96, and ROFA120 groups. ΔP1 augmented early (6 h) in the course of lung injury (184%) and remained elevated until 96 h (137%). ΔP2, Est and ΔE increased in ROFA24 (70, 88, and 68% respectively) and remained elevated until 96 h (68, 50, and 64% respectively). On the fifth day (ROFA120) all parameters returned to CTRL values (Figures 1, 2).

**Figure 3:**
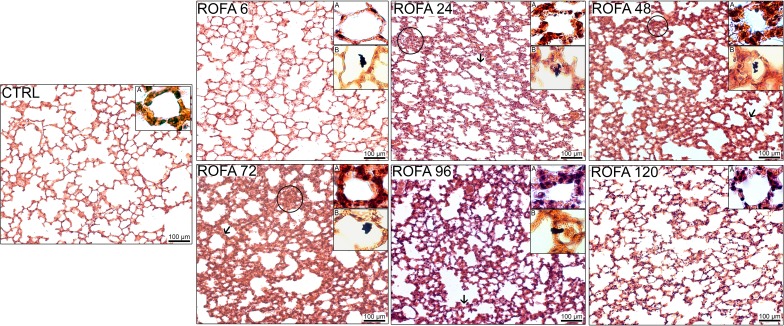
**Photomicrographs of lung parenchyma stained with hematoxylin–eosin (x200)**. CTRL, mice instilled with saline solution (0.9% NaCl, measurements were done 6 h after exposure). ROFA, animals that received residual oil fly ash (0.2 μg in 10 μL of saline solution). Measurements were done 6, 24, 48, 72, 96, and 120 h after exposure. Arrows show representative thickened septa and circles indicate collapsed alveoli. In each panel insert A shows alveolar walls and inflammatory cells therein (×1000 magnification) and insert B displays ROFA particle in the alveolar space when present (×400 magnification). Bar: 100 μm.

**Figure 4:**
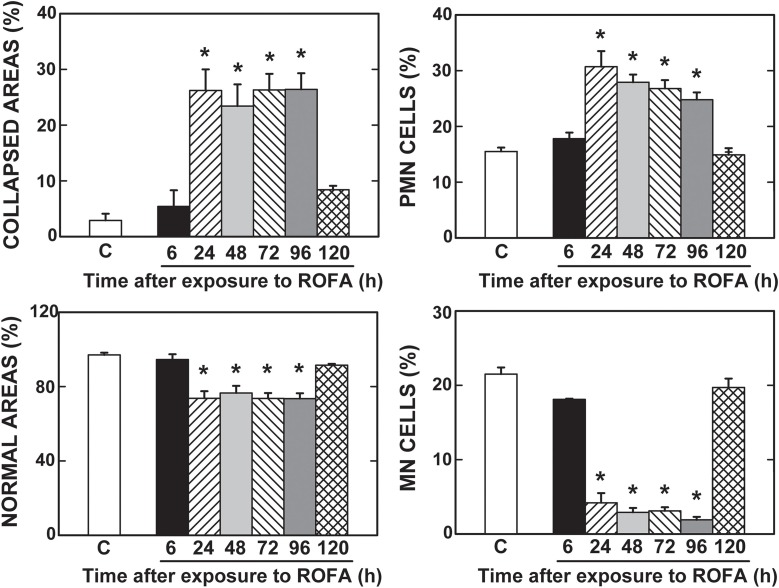
**Collapsed and normal areas, and influx of polymorphonuclear (PMN) and mononuclear (MN) cells**. CTRL, mice instilled with saline solution (0.9% NaCl, measurements were done 6 h after exposure), and ROFA, animals that received residual oil fly ash (0.2 μg in 10 μL of saline solution). Measurements were done 6, 24, 48, 72, 96, and 120 h after exposure. Columns represent the average of 6 mice in each group. Bars represent SEM. Data were gathered from ten random, non-coincident fields per mouse. ^*^Significantly different from CTRL (*p* < 0.05).

The fraction area of alveolar collapse and PMN cell influx into the lung parenchyma were higher in ROFA than in CTRL at 24, 48, 72, and 96 h. Similarly, normal alveolar spaces and MN cells were significantly lower in the latter groups than in CTRL. ROFA120 showed values similar to CTRL for histological parameters (Figures 3, 4). ROFA particle was observed in alveolar space of ROFA6, 24, 48, 72, and 96 groups and not in ROFA120 (Figure 3, insert B).

**Figure 5:**
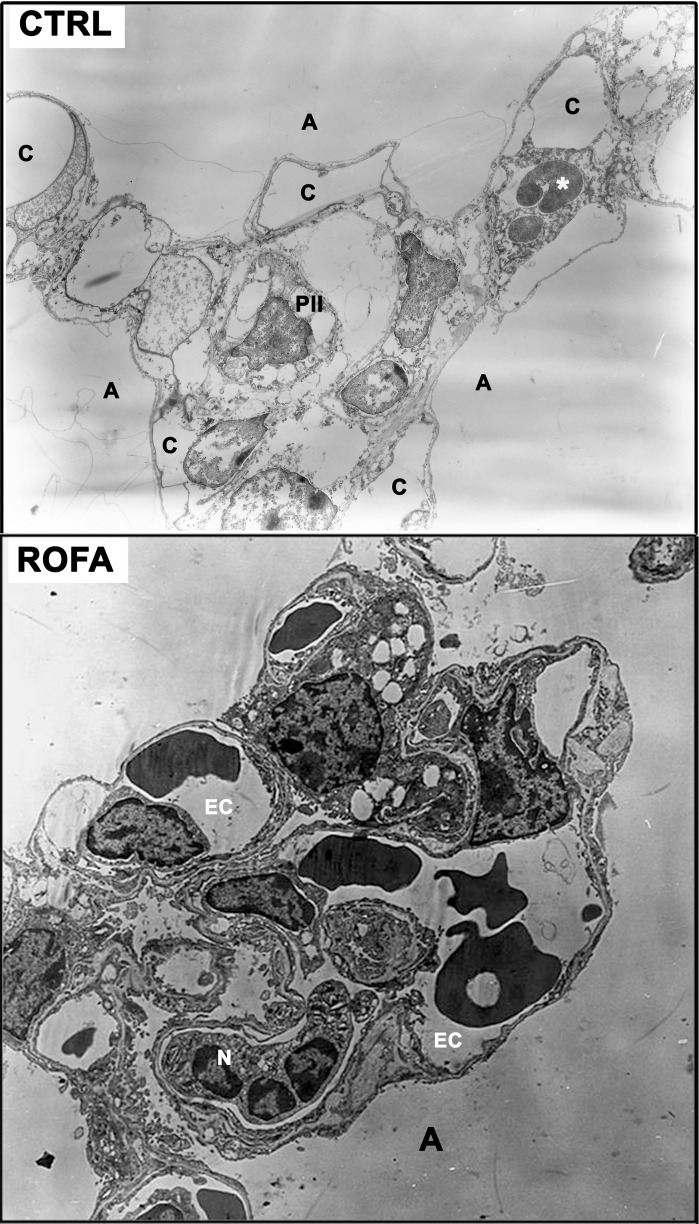
**Electron microscopy of lung parenchyma. Upper panel:** CTRL (6625×), mouse instilled with saline solution. Note the preserved type II pneumocyte (PII) and the alveolar interstitial wall. ^*^Red blood cell. **Lower panel:** ROFA (8400×), animal that received residual oil fly ash (0.2 μg in 10 μL of saline solution). N, adherent neutrophils; C, capillary; A, alveolar space; EC, endothelial cell. Measurements were done 6 (CTRL) and 24 h (ROFA) after exposure.

Electron microscopy of lung parenchyma in CTRL mice showed preserved types I and II pneumocytes, endothelial cells, alveolar interstitial wall, and components of the extracellular matrix. At 24 h, ROFA group showed endothelial damage as represented by swelling, vacuolization and neutrophils adhered to the pulmonary capillary wall (Figure 5).

**Figure 6:**
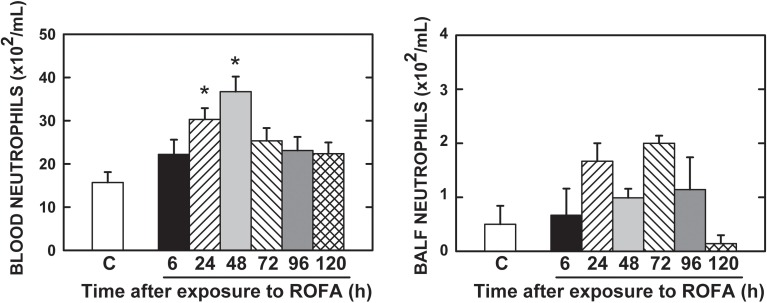
**Neutrophils in the bloodand bronchoalveolar lavage fluid (BALF)**. CTRL, mice instilled with saline solution (0.9% NaCl, measurements were done 6 h after exposure), and ROFA, animals that received residual oil fly ash (0.2 μg in 10 μL of saline solution). Measurements were done 6, 24, 48, 72, 96, and 120 h after exposure. Columns represent the average of 6 mice in each group. Bars represent SEM. ^*^Significantly different from CTRL (*p* < 0.05).

The amount of neutrophils in the blood was higher in ROFA24 and ROFA48 than in the CTRL mice. Thereafter they did not differ from CTRL animals. No statistically significant difference was detected for neutrophils in the BALF (Figure 6).

The survival rate was 100% in all groups throughout the experiments.

## Discussion

The time course of lung functional and histological impairment induced by ROFA dust has not been reported so far. Intranasal instillation of a low dose of ROFA (0.2 μg in 10 μL) induced a significant increase in resistive pressure, followed by an increment in viscoelastic/inhomogeneous pressures and elastances, accompanied by increased alveolar collapse, influx of PMN cells, ultrastructural alterations in lung parenchyma and increased number of neutrophils in the blood. These outcomes returned to control values at 120 h after exposure.

PM is a heterogeneous mixture of gas, liquid, and solid particles of different origins and sizes in suspension in the air, displaying close physical and chemical interactions. PM is classified, according to its aerodynamic diameter, as coarse (2.5–10 μm; PM10), fine (0.1–2.5 μm; PM2.5), and ultrafine (≤0.1 μm) (Donaldson et al., 2001). The different profiles of size and composition may influence particle toxicity and, consequently, the magnitude of adverse health effects (Saldiva et al., 2002). In human beings, toxicity becomes very important when aerodynamic diameter of the particles is 10 μm, which enables them to reach the pulmonary alveoli. In rats and mice, this value approximates 2 μm for intratracheally instilled silica (Wiessner et al., 1989; Takayoshi et al., 2007). The ROFA used in the present study was mainly composed of particles bigger than 10 μm (Avila et al., 2011), which would be less harmful than the smaller ones (Donaldson et al., 2001). However, around 7.6% of ROFA particles presented an average diameter less than 10 μm, and about 2.1% were smaller than 2.5 μm. Another concomitant study of our group (Avila et al., 2011), using the same dose and PM, reported lung impairment at 24 h after exposure. It should be stressed that the fine and ultrafine particles are known as “breathable” and are able to penetrate the airways, reaching the alveoli (Dusseldorp et al., 1995; Peters et al., 1997; Brown et al., 2002; Tao et al., 2003). Indeed, in ROFA6, 24, 48, 72, and 96 groups the administered pollutant was detected in the alveolar space (Figure 3).

A recent study analyzed the composition, sources and toxicity of PM2.5 collected in different cities in the United States and observed an association between its toxicity and the number of vehicles and industries (Seagrave et al., 2006). São Paulo is the most industrialized center of Latin America and has about 20,000,000 inhabitants. There are about 7,000,000 vehicles in the area using three types of fuel: gasoline, diesel and alcohol (CETESB, 2013). Because of its geographical characteristics, São Paulo presents thermal inversions, resulting in significant increases in air pollution. Thus, São Paulo represents an excellent place to assess the effects of air pollution on health.

In order to avoid the consequences of particle overload, we administered a low dose of PM to the mice, reflecting more precisely the adverse pulmonary consequences of ambient particle concentrations. The mean daily concentrations of PM2.5 and PM10 in São Paulo, where ROFA was collected, amount to 60 and 120 μg/m^3^, respectively (CETESB, 2013). Considering that a mouse inspires 0.03 m^3^ of air in 24 h, it represents 6.7 μg/m^3^ of ROFA dust in the present work. Particles were administered by intranasal instillation to the anesthetized animal, which is a useful and well-accepted model of exposure to PM (Southam et al., 2002). In experimental models similar to ours, which used ROFA instilled intranasally in mice, doses amounting to 25 times (Biselli et al., 2011; Magnani et al., 2013; Marchini et al., 2014) and up to 60 times greater (Arantes-Costa et al., 2008) than ours were used.

ROFA is a suspension of the material produced after oil burning, which was used in some experimental models of exposure to air pollution (Ghio et al., 2002; Arantes-Costa et al., 2008; Avila et al., 2011; Marchini et al., 2014). Although ROFA exposure does not exactly mimic the overall environmental pollution, this PM contains high concentrations of many components of air pollution. Previous studies report that PMs from different sources, including ROFA (Kodavanti et al., 1998), are able to induce inflammatory processes (Sørensen et al., 2003; Park et al., 2006). Animal studies demonstrate that the bioavailability of soluble transition metals is responsible for the pulmonary injury and inflammation observed after ROFA exposure (Dreher et al., 1997; Kodavanti et al., 1998). The ROFA used in the present work contains predominantly iron and nickel (Table 1), in line with other studies. Metals, including iron, vanadium, and nickel, are present in high concentrations as water-soluble salts in fly ash (Schroeder et al., 1987) and largely reproduce the lung injury induced by ROFA. Without those metals pulmonary toxicity decreases (Dreher et al., 1997). ROFA containing iron, aluminum, vanadium and nickel induced more pronounced cellular oxidative imbalance and lung injury (Lewis et al., 2003). Iron is deeply linked to the generation of reactive oxygen species (ROS) (Park et al., 2006), surfactant dysfunction (Chauhan and Misra, 1991), epithelial damage, increased vascular permeability and inflammatory response followed by impaired pulmonary function (Soukup et al., 2000; Dye et al., 2001).

In the present study, PM concentration of PAH, particularly naphthalene, acenaphthylene, acenaphthene and other elements with potential carcinogenic risk (benzo[a]antracene, benzo[a]pyrene, Dbenzo[ah]antracene and ind[123cd]pyrene) were detected (Table 2). Another study of our group, analyzing particles produced by traffic, detected benzo[a]pyrene and benzo[a]anthracene in samples of total suspended PM from São Paulo, confirming the high levels of PAH in this city (Mazzoli-Rocha et al., 2008). Washing the diesel particles with hexane removed a large amount of PAHs and improved respiratory outcomes in mice (Laks et al., 2008). Finally, the presence of PAH in ROFA has been associated with the triggering of inflammation, generation of ROS, and lipid peroxidation (Sørensen et al., 2003), especially in alveolar macrophages and epithelial cells (Li et al., 2002).

ROFA was tested for the presence of some organochlorine compounds, and a few were found: op'-DDE, pp'-DDT, op'DDT, G-HCH, endosulfan and dieldrin (Table 3). These substances are pesticides and constitute a family of persistent, lipophilic compounds whose use was banned because they cause a variety of diseases in humans and wildlife (Androutsopoulos et al., 2013). The chlorinated pesticides may be absorbed into the body through the skin, respiratory and digestive tracts (Yohannes et al., 2014). Organochlorines cause neurotoxic, hormonal, immuno-modulating, and tumorigenic effects (Androutsopoulos et al., 2013). However, to our knowledge, no study evaluated the association between exposure to organochlorines and pulmonary impairment.

Although most of lung changes in mechanical properties, histology and inflammatory response occured at time point of 24 h, our control group was studied at 6 h after exposure. To support our approach, we compared our CTRL group with that previously reported and measured at 24 h after exposure to ROFA (Avila et al., 2011), Their results are very similar to ours, thus allowing the use of a 6-h CTRL group. Furthermore, we coped with Russel and Burch's (1959) principle of the 3Rs (reduction, refinement, and replacement of the animal use) to minimize the number of experimental animals.

A higher resistive pressure (that reflects Newtonian or ohmic resistance) was the first response to ROFA, as found in ROFA6 group (Figure 1). It can be possibly explained anatomically, since central airways are the first lung structure to be exposed to ROFA. In a previous study, we also found increased central airway resistance 24 h after exposure to ROFA; it should be noted that the authors did not perform any measurements before that time point (Avila et al., 2011). Viscoelastic and total pressures, static elastance and elastic component of viscoelasticity increased significantly in ROFA24 group and remained elevated until 4 days after exposure (ROFA96), when compared to CTRL (Figures 1, 2). We also detected higher Est and mechanical parameters related to the lung periphery 24 h after exposure to ROFA (Avila et al., 2011). These results could be explained by the concomitant increase in alveolar collapse and lung PMN content (Figures 3, 4). At the same time neutrophils adhered to the swollen pulmonary capillary wall (Figure 5), indicating activation of the endothelium and of leukocyte integrins (Langer and Chavakis, 2009), as a result of a local proinflammatory stimulus presumably triggered by ROFA. These neutrophils would migrate through the endothelium and reach the pulmonary interstitial space (Figure 4). ROFA24 also presented a higher count of PMN in the blood (Figure 6), suggesting a systemic inflammatory status. All these findings were also present in ROFA48 mice. In line with our results in ROFA24 mice, some authors observed impaired lung mechanics, alveolar collapse, influx of inflammatory cells to the lung (Avila et al., 2011), inflammatory process in the perivascular area, and inflammatory infiltration in the interstitial space (Medeiros et al., 2004). Interestingly, a recent study demonstrated increased TNF-α and IL-6 plasma levels and PMN leukocytes activation at 1, 3, and 5 h after ROFA exposure (Marchini et al., 2014), but they used doses 25 times larger than ours. Finally, it should be mentioned that surfactant secretion by type II pneumocytes is impaired after inhalation of air pollutants (Müler et al., 1998) and exposure to fly ash modifies surfactant composition (Srivastava and Misra, 1986; Chauhan and Misra, 1991) and rheology (Anseth et al., 2005), yielding alveolar instability and collapse. At 72 h after exposure blood PMN count returned to control values, but the pulmonary parameters remained higher than CTRL, suggesting that the overall process started to recede in the organism. ROFA96 presented similar results. At 120 h all measured parameters returned to baseline values.

Neutrophils count in the BALF resulted negative (Figure 6). The method may be not sensitive enough to detect the inflammatory changes after the nasal instillation of ROFA or the cells indeed did not cross the airway epithelium. In accordance with our findings, the intranasal instillation of ROFA did not disclose inflammatory alterations in mice BALF, even taking into consideration the use of a dose 500 times larger than ours (Medeiros et al., 2004). On the other hand, the intratracheal instillation of ROFA in a dose 60 times larger than that in this study triggered inflammatory alterations in BALF (Gavett et al., 1999). The difference between these two apparently discrepant results could be the local of administration of the pollutant.

Epidemiological studies can add translational information to our findings. Dose-dependent decreased indexes of pulmonary function, including diminished forced vital capacity, forced expiratory volume in 1 s, and forced expiratory flows were described in boilermakers 24 h after exposure to ROFA (Hauser et al., 1995, 1996). In a 2-year longitudinal study a significant association between working at oil-fired industries and reduced lung function was detected (Hauser et al., 2002). Finally, ROFA-exposed individuals presented impaired pulmonary function, which was resolved 4 weeks after they were removed from their working stations in an oil-fired electricity generating plant (Lees, 1980).

Our study presents some limitations: (1) the animals were exposed intranasally rather than directly to environmental air. On one hand they received only ROFA, but on the other one the results do not represent exactly what would be found around the sampling site; (2) we did not measure levels of inflammatory cytokines that could have been modified by exposure to ROFA.

In conclusion, we demonstrated that the exposure to low doses of ROFA rapidly compromised pulmonary mechanics and histology, triggered the influx of polymorphonuclear cells into the lung, and increased the neutrophil count in the blood of mice. These pathophysiological findings resolved 5 days after exposure.

## Author contributions

Giovanna Marcella Cavalcante Carvalho—interpretation of data for the work; drafting the work and revising it for important intellectual content; final approval of the version to be published; agreement to be accountable for all aspects of the work in ensuring that questions related to the accuracy or integrity of any part of the work are appropriately investigated and resolved; Lilian Katiê da Silva Nagato—interpretation of data for the work, experimental design and organization, data analyses; revised the work for important intellectual content; final approval of the version to be published, agreement to be accountable for all aspects of the work in ensuring that questions related to the accuracy or integrity of any part of the work are appropriately investigated and resolved; Sheila da Silva Fagundes—data acquisition; revised the work for important intellectual content; final approval of the version to be published; agreement to be accountable for all aspects of the work in ensuring that questions related to the accuracy or integrity of any part of the work are appropriately investigated and resolved; Flávia Brandão dos Santos—data acquisition; revised the work for important intellectual content; final approval of the version to be published; agreement to be accountable for all aspects of the work in ensuring that questions related to the accuracy or integrity of any part of the work are appropriately investigated and resolved; Andrea Surrage Calheiros—data acquisition; revised the work for important intellectual content; final approval of the version to be published; agreement to be accountable for all aspects of the work in ensuring that questions related to the accuracy or integrity of any part of the work are appropriately investigated and resolved; Olaf Malm—chemical analyses; revised the work for important intellectual content; final approval of the version to be published; agreement to be accountable for all aspects of the work in ensuring that questions related to the accuracy or integrity of any part of the work are appropriately investigated and resolved; Patricia Torres Bozza—data analyses; revised the work for important intellectual content; final approval of the version to be published; agreement to be accountable for all aspects of the work in ensuring that questions related to the accuracy or integrity of any part of the work are appropriately investigated and resolved; Paulo Hilário N. Saldiva—data analyses; revised the work for important intellectual content; final approval of the version to be published; agreement to be accountable for all aspects of the work in ensuring that questions related to the accuracy or integrity of any part of the work are appropriately investigated and resolved; Débora Souza Faffe—experimental design and organization; revised the work for important intellectual content; final approval of the version to be published; agreement to be accountable for all aspects of the work in ensuring that questions related to the accuracy or integrity of any part of the work are appropriately investigated and resolved; Patricia Rieken Macedo Rocco—experimental design and organization; revised the work for important intellectual content; final approval of the version to be published; agreement to be accountable for all aspects of the work in ensuring that questions related to the accuracy or integrity of any part of the work are appropriately investigated and resolved; Walter Araujo Zin—experimental design and organization, hypotheses, interpretation of data for the work; drafting the work, revised the work for important intellectual content; final approval of the version to be published, agreement to be accountable for all aspects of the work in ensuring that questions related to the accuracy or integrity of any part of the work are appropriately investigated and resolved.

## Support

Centers of Excellence Program (PRONEX/FAPERJ), The Brazilian Council for Scientific and Technological Development (CNPq), Carlos Chagas Filho Rio de Janeiro State Research Supporting Foundation (FAPERJ), Brazilian Ministry of Science, Technology and Innovation (MCTI), and Financing for Studies and Projects (FINEP). The funders had no role in study design, data collection and analysis, decision to publish or preparation of the manuscript.

### Conflict of interest statement

The authors declare that the research was conducted in the absence of any commercial or financial relationships that could be construed as a potential conflict of interest.
